# A Study of the Interaction, Morphology, and Structure in Trypsin-Epigallocatechin-3-Gallate Complexes

**DOI:** 10.3390/molecules26154567

**Published:** 2021-07-28

**Authors:** Jiayin Liu, Hossein Ghanizadeh, Xinmao Li, Zhengyuan Han, Youwen Qiu, Yao Zhang, Xiuling Chen, Aoxue Wang

**Affiliations:** 1 College of Horticulture and Landscape Architecture, Northeast Agricultural University, Harbin 150030, China; liujiayin@neau.edu.cn (J.L.); lixinmao2020@163.com (X.L.); hzy0226@126.com (Z.H.); chenx@neau.edu.cn (X.C.); 2 College of Arts and Sciences, Northeast Agricultural University, Harbin 150030, China; 3 School of Agriculture and Environment, Massey University, 4410 Palmerston North, New Zealand; H.GhaniZadeh@massey.ac.nz; 4 College of Life Sciences, Northeast Agricultural University, Harbin 150030, China; yw12_630@126.com (Y.Q.); yaoyao705116358@sina.com (Y.Z.)

**Keywords:** trypsin, EGCG, aggregates, small-angel X-ray scattering, polyphenols

## Abstract

Understanding the interaction between proteins and polyphenols is of significance to food industries. The aim of this research was to investigate the mode of aggregation for trypsin-EGCG (Epigallocatechin-3-gallate) complexes. For this, the complex was characterized by fluorescence spectroscopy, circular dichroism (CD) spectra, small-angel X-ray scattering (SAXS), and atomic force microscope (AFM) techniques. The results showed that the fluorescence intensity of trypsin-EGCG complexes decreased with increasing the concentration of EGCG, indicating that the interaction between trypsin and EGCG resulted in changes in the microenvironment around fluorescent amino acid residues. The results of CD analysis showed conformational changes in trypsin after binding with EGCG. The results from SAXS analysis showed that the addition of EGCG results in the formation of aggregates of trypsin-EGCG complexes, and increasing the concentration of EGCG resulted in larger aggregates. AFM images showed that the trypsin-EGCG complex formed aggregates of irregular ellipsoidal shapes with the size of about 200 × 400 × 200 nm, with EGCG interconnecting the trypsin particles. Overall, according to these results, it was concluded that the large aggregates of trypsin-EGCG complexes are formed from several small aggregates that are interconnected. The results of this study shed some light on the interaction between digestive enzymes and EGCG.

## 1. Introduction

Polyphenols are naturally occurring organic compounds with antioxidant, anti-inflammatory, and anticancer activities and are present in plant-based foods and beverages [1,2]. They constitute a large group of bioactive phytochemicals, such as flavonoids, phenolic acids, stilbenes, and lignans [3]. Thousands of polyphenol compounds have been identified in higher plants, with hundreds of them found in edible plants. Polyphenols are abundant in tea, cocoa, fruits, berries, and vegetables [4]. Polyphenols can prevent the negative effects of reactive oxygen species and reactive nitrogen species, ultraviolet rays, plant pathogens, parasites, and predators to a certain extent, which leads to some beneficial biological activities, such as the prevention and even treatment of some popular human diseases, especially various types of cancer [5]. As secondary plant metabolites, polyphenol compounds have a role in defense against ultraviolet radiation and are involved in defense against different types of stress [6,7]. In food, polyphenols can contribute to the flavor, color, and oxidative stability [8].

Polyphenols can interact with proteins and form polyphenol-protein complexes [9]. The formation of polyphenol-protein complexes is of immense importance in the field of medicine, the food industry, and human health [10,11]. The formation of polyphenol-protein complex can affect the structure of proteins and, subsequently, the activity of the enzymes. Meanwhile, binding to proteins may affect the absorption of polyphenol compounds and reduces their bioavailability and nutritional benefits [12]. Factors such as the structures of polyphenols and proteins and the ratio of polyphenol/protein can affect the mode of interaction between proteins and polyphenols [13]. The interaction between polyphenols and several enzymes such as tyrosinase, amylase, xanthine oxidase, and peroxidase has been reported [14]. Polyphenols can also interact with digestive enzymes; however, the interaction between digestive enzymes and polyphenols at molecular levels and the mechanism of the aggregating complex have rarely been investigated. Thus, determining the molecular basis of interaction between polyphenols and proteins is of great significance.

Epigallocatechin-3-gallate (EGCG) (Figure S1 in Supplementary Materials) is the ester of epigallocatechin and gallic acid, which possesses antioxidant activity and can be found in abundance in green tea [2]. EGCG can bind to various proteins, including trypsin, which is a digestive enzyme with protease activity [15]. Binding EGCG to trypsin results in the formation of soluble or insoluble complexes [3,4]. Studying the interaction between EGCG and trypsin provides a new perspective for understanding the role of protein-polyphenol complexes in the digestive system [16]. Thus, the objective of this research was to characterize the interaction between trypsin and EGCG, and to study the binding properties of the trypsin-EGCG complex at a molecular level. For this, the binding constant and binding sites of the trypsin-EGCG complex were estimated using the fluorescence spectra method. The structural changes of trypsin were also investigated after binding to EGCG using the small-angle X-ray scattering (SAXS) method. SAXS is a unique high-throughput method for characterizing the structure of macromolecules. The advantages of this method are that the samples require minimal preparation, and the protein can be assessed under near-physiological conditions [17]. SAXS has widely been used for investigating the protein interactions, protein conformational changes, and the formation/destruction of high-order complexes [18]. In this research, the effects of the aggregation process on the conformation and activity of trypsin were eventually studied.

## 2. Materials and Methods

### 2.1. Materials

Trypsin was purchased from Sigma Aldrich (Saint Louis, MO, USA) and stored at −20 °C. EGCG was purchased from Alatin (Shanghai, China). Tris-HCl and other chemicals were of analytical grade or higher. Distilled water was used in all experiments.

### 2.2. Preparation of Trypsin-EGCG Complexes

The trypsin stock solution was prepared by dissolving trypsin in PBS buffer (phosphate-buffered saline, 0.02 mM, pH = 7.4), and the final concentration of the prepared solution was 16.8 μM. The EGCG stock solution was prepared by adding EGCG into the same PBS buffer, and the final concentration of the prepared solution was 1.396 mM. In order to assess the effect of different concentrations of EGCG, some sets of samples were prepared by adding various amounts of the EGCG solution to the trypsin solution. The samples were stirred vigorously at room temperature. Throughout the experimental procedures, the final concentration of trypsin was maintained constant (0.8 mg/mL), while the concentration of EGCG varied from 0 to 0.0016 mg/mL. The resultant mixtures of trypsin and EGCG were characterized by the method outlined previously [19].

### 2.3. Evaluating the Secondary Structure Changes of Trypsin

The changes in the secondary structure of trypsin in the presence of EGCG were assessed using the circular dichroism (CD) method. CD spectra were recorded on a Jasco J-810 spectropolarimeter (Tokyo, Japan). The concentration of trypsin was kept at 8.4 μM, while the concentrations of EGCG were varied from 0~698 μM. The samples were scanned from 190 to 260 nm, and spectra were recorded at a speed of 200 nm min^−1^. Three scans were performed and averaged for each spectrum.

### 2.4. Evaluating the Binding Affinity between Trypsin and EGCG

The binding affinity between trypsin and EGCG was assessed using a fluorescence method. Fluorescence spectra were obtained by a fluorescence spectrophotometer (F-7000, Hitachi, Tokyo, Japan). The EGCG solution was added to the trypsin solution, and the final volume was adjusted to 1 ml. The PBS buffer was used as the background. The fluorescence spectra were recorded at λ_exc_ = 280 nm and λ_em_ from 300 to 540 nm. The modified Stern–Volmer Equation (1) was used to calculate the binding constant (K) and the binding affinity (∆G).

lg(F_0_ − F)/F = lgKa + nlg[Q] (1)

where Ka represents the binding constant, F_0_/F represents the fluorescence intensities in the presence and absence of EGCG, respectively [20]. The excitation and emission slit width were both set at 4 nm. All the experiments were repeated three times.

### 2.5. Structural Changes in Trypsin-EGCG Complexes

The structural changes in trypsin-EGCG complexes were assessed using the SAXS method. The SAXS experiments were performed at the BioCAT Beamline 18-ID at the Advanced Photon Source, Argonne National Laboratory, Lemont, IL, USA. The wavelength of X-ray radiation was 1.033 Å, and a short exposure period of 1 s was used to acquire the scattering data. SAXS measurements were performed according to the procedure outlined previously [21]. The final SAXS profiles were obtained through an average of 15 measurements followed by the subtraction of solvent background. GNOM and CRYSOL were used to compute simulated scattering curves.

### 2.6. Evaluating the Protein-Ligand Systems

The aggregation state of trypsin-EGCG complexes was evaluated by atomic force microscopy (AFM) images [22]. The trypsin-EGCG solution was placed on silica and dried in an N_2_ atmosphere and analyzed immediately. AFM images were performed by an Agilent 5500 atomic force microscope (Keysight, Santa Rose, CA, USA). We used NTESP silicon cantilevers (Bruker, Madison, WI, USA) with a typical resonant frequency of 300 kHz and a nominal tip radius of 10 nm. Images were acquired at a scan rate of 1 Hz. The images were taken at least five times at different parts of the complex particles.

## 3. Results and Discussion

### 3.1. Analysis of Fluorescence Spectroscopy Data

**Figure 1:**
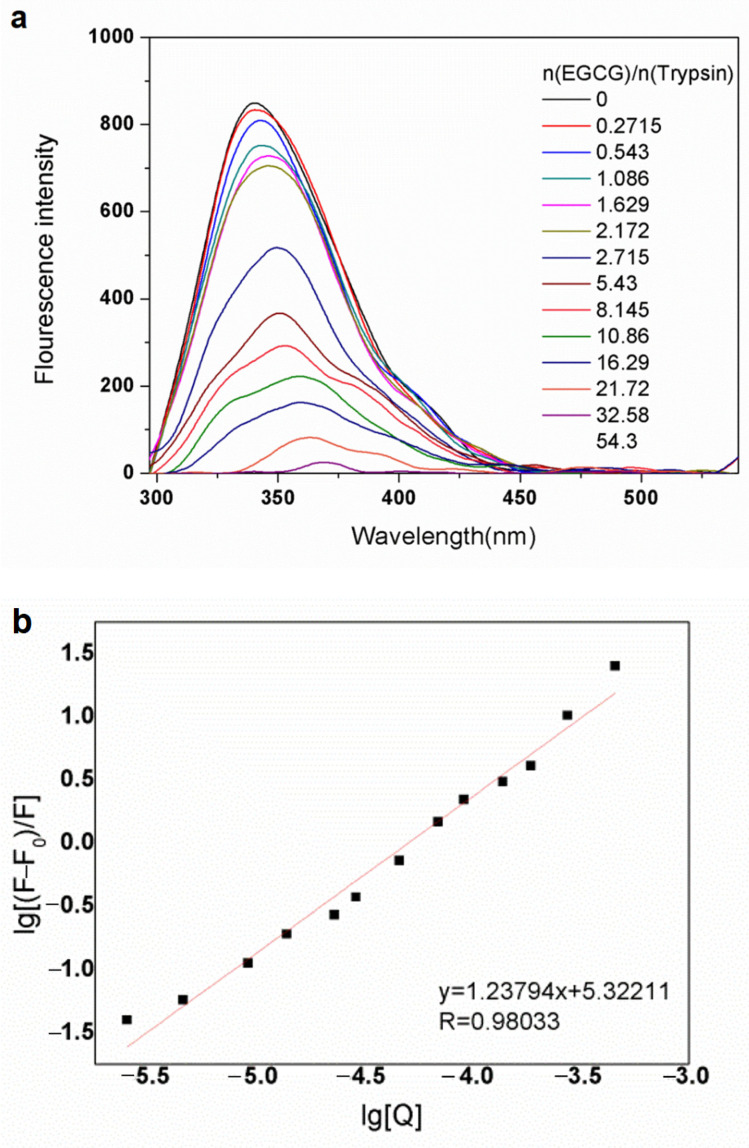
(**a**) Quenching effect of EGCG on trypsin fluorescence intensity as a function of EGCG concentrations. (**b**) The relationship between different concentrations of EGCG and fluorescence intensity can be described by the modified Stern–Volmer equation.

The interaction between trypsin and EGCG was determined by the fluorescence spectrometry method. Trypsin has an inherent fluorescence property, and its fluorescence property is mainly dominated by ten tyrosine and four tryptophan [23]. According to Figure 1a, the fluorescence intensity of trypsin-EGCG complexes decreased with increasing the concentration of EGCG, indicating that the interaction between trypsin and EGCG resulted in changes in the microenvironment around fluorescent amino acid residues. No changes in the fluorescence intensity of treatments containing EGCG only was recorded. However, the binding of EGCG to trypsin changed the microenvironment of the chromophore of trypsin. Binding EGCG to trypsin might also have changed the protein chain via forming new hydrogen bonds; thus, it resulted in the reduction in the fluorescence intensity of the protein-polyphenol complex. Previous research showed that EGCG could bind to the primary substrate-binding pocket and change the microenvironment around Trp and Tyr [20]. It is possible that EGCG entered the substrate-binding pocket of trypsin and resulted in a reduction in the fluorescence intensity.

The relationship between different concentrations of EGCG and fluorescence intensity can be described by the modified Stern–Volmer equation (Figure 1b). The equation was used to calculate the binding constant (Ka), binding sites (n), and Gibbs free energy (∆G). The results showed that there was a linear relationship between different concentrations of EGCG and fluorescence intensity, as shown by a high R-value. According to the results, an n value of 1.23 was estimated, indicating that the binding ratio of EGCG to trypsin was about 1:1. The number of binding sites for each trypsin molecular was 1.23, with a lgKa value of 5.3211. The results also showed that there was a binding constant value of 1.7 × 10^5^ L mol^−1^ and the value of free energy change was −29.8 KJ mol^−1^ (T = 298 K). The binding constants (Ka) value has a crucial role in understanding the distribution of polyphenols in the stomach or digestive system [24]. A low value of Ka is an indication of better distribution of polyphenols [25]. The negative value of ∆G indicates that the interaction between trypsin and EGCG is spontaneous [26]. Moreover, a slight red shift occurred in fluorescence intensity, suggesting that the trypsin molecular was unfolded, and the polarity of the trypsin environment was increased [27]. It is well known that the fluorescence quenching process of trypsin in the presence of EGCG is governed by a static quenching mechanism rather than a dynamic quenching process. The quenching effect mostly depends on the C-ring structure of the polyphenols [28].

### 3.2. SAXS Measurements

**Figure 2:**
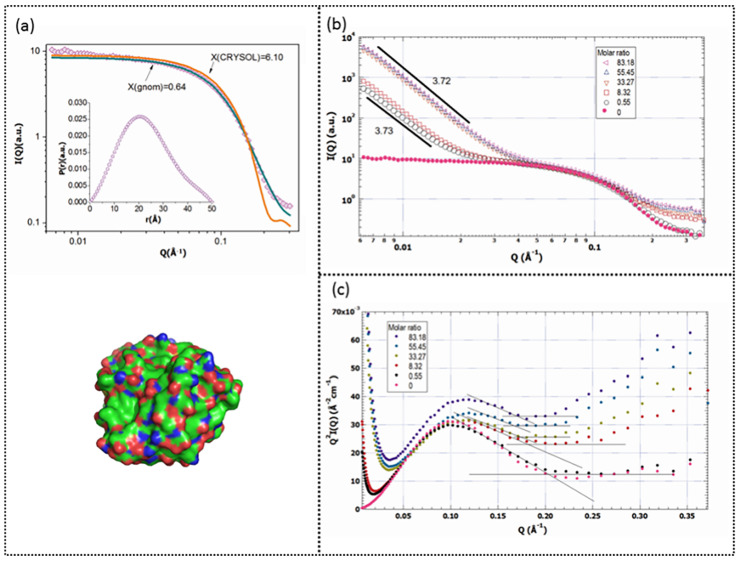
(**a**) The intensity profiles from SAXS of trypsin in 1 mg/mL solution and the PDB photo of trypsin. The inset shows the pair distance distribution function. (**b**) The scattering intensity profiles of trypsin-EGCG complex with different concentrations of EGCG. (**c**) The Kratky plot of the trypsin-EGCG complex as a function of the molar ratios of EGCG/trypsin (the concentration of trypsin was fixed at 1 mg/mL).

The results for the SAXS analysis of the trypsin-EGCG complex are shown in Figure 2. As shown in Figure 2a, the curves of samples were fitted to a spherical model using GNOM and CRYSOL programs. According to the results, GNOM fit yielded a root mean square deviation (χ^2^) of 0.64, which was lower than that of CRYSOL (6.10). The pair distance distribution function (P(r)) in GNOM fit confirms the spherical shape of trypsin molecules in the system. The scattering intensity profiles of different samples at the intermediate q-range suggested that trypsin still kept a monomer status in the complex (Figure 2a).

**Figure 3:**
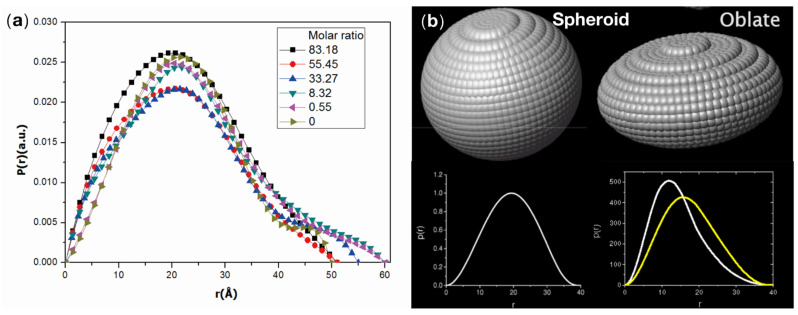
(**a**) Distance distribution functions from the complexes. (**b**) The molecular shape of trypsin at low (right) versus high (left) concentrations of EGCG.

According to Figure 2b, there were different SAXS patterns for trypsin-EGCG complex samples compared to the trypsin-only sample. The plot of trypsin in the absence of EGCG revealed linearity in the small q region, while in the same region, the presence of EGCG increased the scattering intensity. The sharp increase in scattering intensity at the low range of q indicates a large-scale aggregation of the trypsin-EGCG complex. Therefore, it could be concluded that aggregates were formed after EGCG addition and increasing the concentration of EGCG resulted in larger aggregates. Furthermore, the fractal dimension (Df) at the low range of q is about 3.7, which is close to the value for the Porod smooth surface fractal dimension (4.0) [29]. These results indicate that the surface of the trypsin- EGCG complex monomer is large, dense, and smooth. The Kratky plot (Figure 2c) indicates the folding state of trypsin under different EGCG concentrations. The Kratky plots of the SAXS exhibited a symmetrical peak, indicating a globular shape of trypsin, while the plots become linear at the high values of q. Trypsin without EGCG showed its native folding state, and its Kratky plot revealed a pronounced peak with a “bell” shape. If the protein is completely unfolded, the Kratky plot will show an obvious increase at the high values of q [30,31]. The tilted part of the plot showed that in the presence of EGCG, the peptide chain of trypsin became denatured, indicating that EGCG binding caused a partial trypsin unfolding. According to the standard mode, the pair distance distribution function in Figure 3 revealed that each trypsin particle is an oblate (flattened) spheroid. Binding with EGCG of different concentrations had a small effect on the shape of trypsin particle as curves with different molar ratios are similar to each other. As the concentration of EGCG increased, the p(r) function became more asymmetric, and trypsin particles became more spherical and prone to form aggregates.

The radius of gyration (R_g_, G) was estimated from the slope of the patterns (Table 1). The results showed that the R_g_ value changed at different EGCG/trypsin molar ratios, suggesting that trypsin varied in size at different amounts of EGCG. In the absence of EGCG, the R_g_ value of trypsin, in its native state, was 17. The R_g_ value slightly increased from 17.8 to 18.4 before it reduced to 17.3 when EGCG was added. The initial increase in the R_g_ value could be due to partially loosen of the peptide chains after EGCG binding. Once the trypsin-EGCG complex formed, the peptide chain crowned more tightly, so the R_g_ value reduced to 17.3. Overall, the R_g_ value decreased slightly with increasing the concentration of EGCG, indicating that trypsin molecules collapse after EGCG binding.

### 3.3. Conformational Changes in Protein

**Figure 4:**
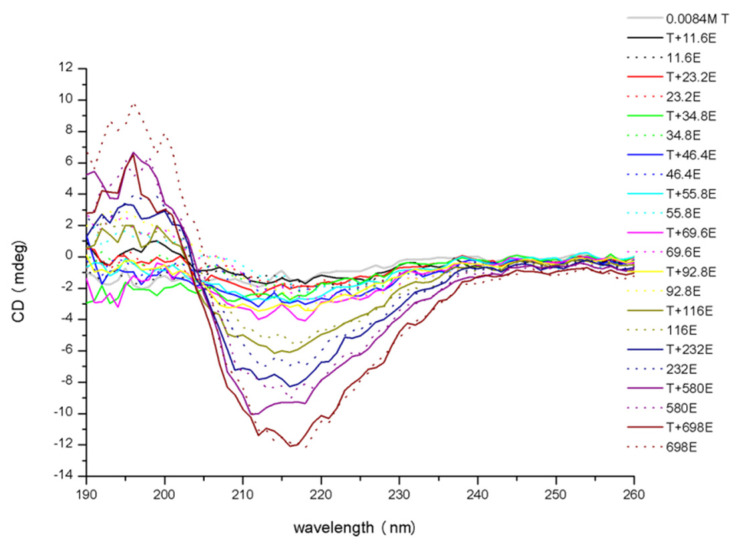
The CD spectra of trypsin in the presence and absence of EGCG.

The results of CD analysis showed conformational changes in trypsin after binding with EGCG (Figure 4). The spectrum of free trypsin showed a negative band in the ultraviolet region at about 215 nm. With the addition of EGCG to trypsin, the negative molar ellipticity of trypsin increased. This indicates that EGCG affected the secondary structures of trypsin, and this can result in changes in the physiological function of trypsin.

### 3.4. AFM Images Measurements

**Figure 5:**
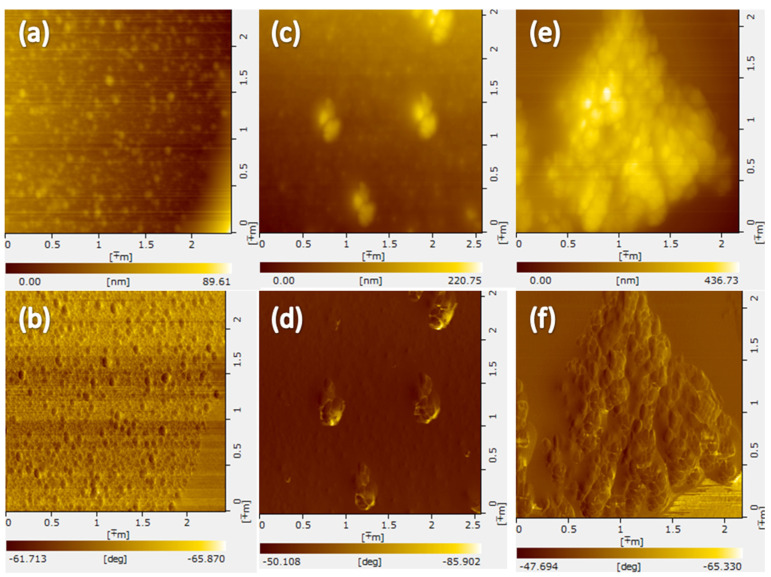
AFM images of the trypsin-EGCG complex at different EGCG concentrations with phase image (**b**,**d**,**f**) and 2D topographic profile (**a**,**c**,**e**); (**a**,**b**) pure trypsin (8.4 μM); (**c**,**d**) the trypsin-EGCG complex (trypsin 8.4 μM, EGCG 30 μM); (**e**,**f**) the trypsin-EGCG complex (trypsin 8.4 μM, EGCG 60 μM).

The results from the AFM images revealed that trypsin particles in the absence of EGCG aggregated into microspheres with a diameter of about 100 nm (Figure 5). At the EGCG concentration of 30 μM, the trypsin-EGCG complex formed aggregates of irregular ellipsoidal shapes with the size of about 200 × 400 × 200 nm (Figure 5c,d). Interestingly, each aggregate was formed by several particles with EGCG molecules acting as bridges connecting the protein particles [32]. Increasing the concentration of EGCG results in the formation of larger aggregates (Figure 5e,f). Similarly, the previous study has suggested that different EGCG/trypsin molar ratios lead to varied morphology of the complexes, and the interaction between EGCG and trypsin is a function of the concentration of EGCG [33]. Non-covalent binding such as the hydrogen bonds, can play an important role in enhancing the binding affinity and the alteration of the conformational structure of trypsin [34]. EGCG contained phenolic groups, which can form hydrogen bonds with the polar groups of trypsin. The addition of EGCG to protein can alter the internal interactions of the protein chains [35] and promotes the aggregation of the protein-EGCG complex [30].

**Figure 6:**
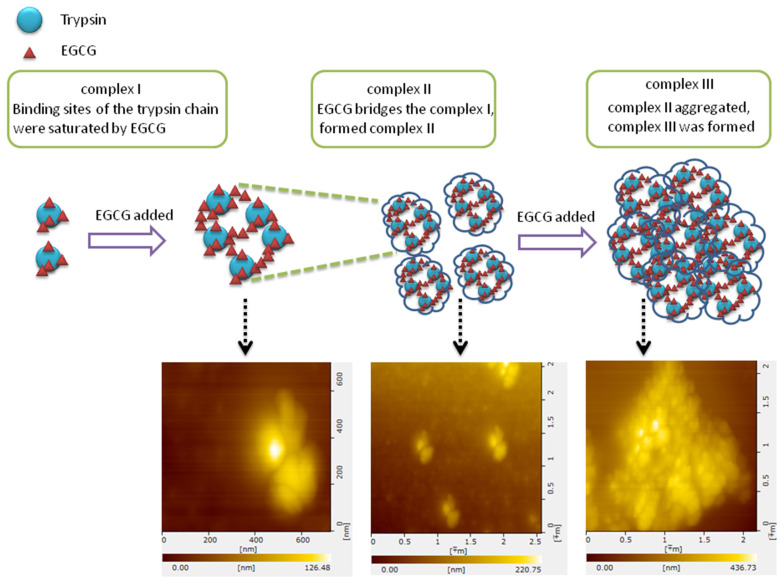
Schematic graph of the aggregate process.

As shown in Figure 6, the formation of the trypsin-EGCG complex consists of three phases. In the first phase, the binding sites of the enzyme is saturated by EGCG, with binding EGCG to the enzyme results in unfolding or conformational changes of trypsin (complex I), as shown by the Rg values from the SAXS analysis in this research. In the second phase, increasing the concentration of EGCG will saturate the biding sites of the enzyme, and the excess EGCG molecules serve as bridges connecting the trypsin molecules and form metastable colloids (complex II). According to the AFM images, the shape of complex II was regular, and the size was about 200 × 400 × 200 nm. In the third phase, with increasing the concentration of EGCG, EGCG-saturated trypsin particles aggregate through EGCG bridges and form haze particles (complex III). Increasing the EGCG/trypsin ratios in the system results in larger complexes in the third phase due to the further aggregation of the complexes. Based on these results, two modes of aggregations can be suggested: (1) the large aggregates are formed from several small aggregates that are interconnected; (2) the large aggregates originated as one small aggregate in the center, which is surrounded by other aggregates. The AFM image of complex II, however, supports the former mode of aggregation, and the shape and size of the aggregates were found to be EGCG concentration-dependent as increasing the concentration of EGCG leads to the formation of larger aggregates.

## 4. Conclusions

The binding properties of trypsin and EGCG and the mode of aggregation for the trypsin-EGCG complex were investigated here. The complex of trypsin and EGCG was characterized by various methods. The results showed that trypsin and EGCG could interact and form a complex, and the morphology and properties of the complex is EGCG concentration-dependent. Overall, the results of this study shed some light on the interaction between digestive enzymes and EGCG.

## Figures and Tables

**Table 1:** The SAXS derived parameters (Rg, Df, lp, and lc) at different molar ratios of EGCG/trypsin.

Molar Ratio (EGCG/Try)	Rg(A)		Df	lp(nm)	lc(Å)
	Gunier	GNOM			
0	17.75	17.41	0.78	32.90	22.23
0.55	18.41	19.00	3.73	31.73	345.38
8.32	18.13	19.13	3.73	34.52	402.72
33.27	17.32	17.98	3.75	36.32	427.90
55.45	17.68	16.80	3.76	37.62	446.19
83.18	17.26	17.17	3.72	36.74	407.41

## Data Availability

All relevant data are within the manuscript.

## Supplementary Materials

The following are available online, Figure S1: Chemical structure of Epigallocatechin-3-gallate. The red arrow indicates the position where the stereochemistry change occurs.

## Sample Availability

Samples of the compounds are available from the authors.
