# Supplementary material for: A Study of the Interaction, Morphology, and Structure in Trypsin-Epigallocatechin-3-Gallate Complexes
Source: Molecules. 2021 Jul 28;26(15):4567. doi: 10.3390/molecules26154567 (PMC8348591; doi:10.3390/molecules26154567)
Supplement: Supplementary file 1 [file molecules-26-04567-s001.zip › molecules-1280552-supplementary.pdf]

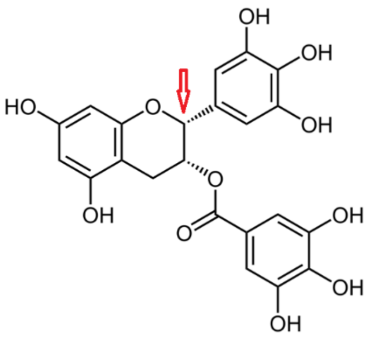

Figure S1. Chemical structure of Epigallocatechin-3-gallate. The red arrow indicates the position where the stereochemistry change occurs.
